# Recent advances in understanding hepatitis C

**DOI:** 10.12688/f1000research.7354.1

**Published:** 2016-02-03

**Authors:** Florian Douam, Qiang Ding, Alexander Ploss

**Affiliations:** 1 Department of Molecular Biology, Princeton University, 110 Lewis Thomas Laboratory, Washington Road, Princeton, NJ, 08544, USA

**Keywords:** hepatitis C, HCV, Anti-viral therapy, pathogenesis, immune response

## Abstract

The past decade has seen tremendous progress in understanding hepatitis C virus (HCV) biology and its related disease, hepatitis C. Major advances in characterizing viral replication have led to the development of direct-acting anti-viral therapies that have considerably improved patient treatment outcome and can even cure chronic infection. However, the high cost of these treatments, their low barrier to viral resistance, and their inability to prevent HCV-induced liver cancer, along with the absence of an effective HCV vaccine, all underscore the need for continued efforts to understand the biology of this virus. Moreover, beyond informing therapies, enhanced knowledge of HCV biology is itself extremely valuable for understanding the biology of related viruses, such as dengue virus, which is becoming a growing global health concern. Major advances have been realized over the last few years in HCV biology and pathogenesis, such as the discovery of the envelope glycoprotein E2 core structure, the generation of the first mouse model with inheritable susceptibility to HCV, and the characterization of virus-host interactions that regulate viral replication or innate immunity. Here, we review the recent findings that have significantly advanced our understanding of HCV and highlight the major challenges that remain.

## Introduction

Hepatitis C virus (HCV) is a single-stranded, positive-sense RNA virus of the *Flaviviridae* family. Although the inability to culture primary HCV isolates *in vitro* seriously hampered HCV research for the 15 years following its isolation in 1989 ^1^, the generation of relevant *in vitro* systems in the early 2000s offered the first opportunities to accurately characterize the HCV life cycle. Since then, considerable progress has been made to understand HCV biology through the generation of increasingly relevant cell culture systems and animal models. These advances recently reached a milestone by the generation of effective direct-acting anti-viral agents (DAAs) ^2^ able to cure HCV.

However, a large number of challenges remain to significantly decrease HCV spread on a global scale. Indeed, 130 to 150 million people worldwide are still chronically infected with HCV (World Health Organization), and treatments remain poorly accessible because of their high costs. Moreover, their low barrier to resistance-associated mutations combined with their inability to treat challenging patient groups, HCV-induced liver disease, and hepatocellular carcinoma (HCC) all underscore the need for novel, cost-effective DAAs ^3– 6^. Finally, a prophylactic or preventive HCV vaccine is still urgently needed to significantly impact HCV spread worldwide.

Overall, these challenges strongly highlight that our understanding of HCV and its related disease remains incomplete and that efforts need to be maintained to expand it. Here, we review the recent advances that have greatly contributed to improving our knowledge of hepatitis C and HCV and highlight the fundamental and clinical challenges that still need to be faced by the HCV scientific community.

## A. Hepatitis C virus life cycle

### 1. Toward a better understanding of the hepatitis C virus entry process

HCV enters into hepatocytes through a dynamic, multi-step process involving multiple cell host factors ^7^. As the primary attachment of viral particles at the hepatocyte surface occurs through interactions with lipoprotein receptors, the association between HCV particles and lipoproteins is critical for initiating the first step of virus entry ^8^. After primary attachment, HCV particles interact with the tetraspanin CD81 via the viral E2 glycoprotein ^9^, which, along with E1, constitutes a heterodimer complex at the surface of viral particles ^7^. E2 interaction with CD81 is thought to induce signaling pathways involving epidermal growth factor receptor (EGFR) ^10, 11^ and HRas ^12^ that together lead to the clustering of CD81-viral particle complexes with the tight junction protein claudin-1 (CLDN1) ^13, 14^. Although direct interaction between HCV particles and CLDN1 was not initially demonstrated, recent evidence supports such interactions ^15, 16^. Another tight junction protein, occludin (OCLN), is also critical for a late step of virus entry ^17, 18^, although its precise role during this process has not been clearly defined. CD81-CLDN1 clustering is thought to induce the internalization of viral particles through clathrin-dependent endocytosis ^19, 20^. The fusion of the viral particle and late endosome membranes, which is thought to be mediated by structural rearrangements of the E1E2 heterodimer complex, then results in the release of viral RNA into the cytosol.

***Uncovering the spatio-temporal dynamics of hepatitis C virus entry.*** A considerable challenge in understanding HCV entry is accurately capturing the spatio-temporal dynamics of this process, as it involves a considerable number of host factors and regulators, both extracellularly and intracellularly. The recent identification of additional factors important for virus entry has only complicated this problem. Among those additional factors are the Niemann-Pick C1-like 1 (NPC1L1) receptor ^21^, the transferrin-1 receptor ^22^, and even more recently, via a proteomic approach, the serum response factor-binding protein 1 (SRFBP1) ^23^. Although these new factors are thought to be important for late steps of virus entry, for rearrangement of lipoproteins within the viral particle, or for CD81-induced signaling pathways, their role in the spatio-temporal dynamics of virus entry still needs to be clearly defined.

***The elusive hepatitis C virus fusion mechanism.*** In addition to viral entry dynamics, the HCV fusion process remains not fully understood because of the absence of crystal structures of E1 and the E1E2 protein complex. It was previously thought that E2 glycoproteins might harbor a class II fusion protein structure ^24^, thus mediating the fusion between the viral and the endosomal membranes in a manner similar to flaviviruses. However, very recently, the structural resolution of the central core of the E2 protein ^25, 26^, E2core, revealed that E2 harbors a globular, non-extended structure that does not display any features of a class II fusion protein. In parallel, this finding has been strengthened by evidence that E1 might function as a fusion protein ^27– 29^, despite unusual N-terminal structural organization ^30^. In the future, structural resolution of E1 and E1E2 complexes at pre- and post-fusion conformational states should unveil critical features of the fusion mechanism. Overall, this process would likely be unique in the *Flaviviridae* family and be mediated by a very original, interdependent interplay between E1 and E2.

### 2. miR-122 and lipid metabolism: key regulators of viral replication and assembly

HCV RNA replication occurs in altered, endoplasmic reticulum (ER)-derived membrane structures known as the “membranous web” (MW). Such structures are known to be critical for RNA replication and have been observed both *in vitro* and *ex vivo* ^31– 34^.

The MW is a complex network of altered membrane structures, formed through the concerted action of several non-structural proteins ^34^. Several lines of evidence suggest that double membrane vesicles (DMVs), which represent the major components of the MW, could represent the sites of viral RNA replication in infected cells. Indeed, the viral proteins NS3 and NS5A and the active viral replicase have been found in DMVs, along with vesicle-associated membrane protein-associated protein A and cholesterol, host factors critical for viral RNA replication ^34, 35^.

During replication, the RNA-dependent RNA polymerase NS5B ensures the production of newly synthetized positive-strand RNA ^31, 36, 37^ following generation of negative-strand RNA. Recently, 12 different structures of NS5B were crystallized during primed initiation or elongation of RNA synthesis, thus providing a unique look at the structural basis of HCV RNA replication and the inhibitory mechanism of nucleot(s)ide-analog inhibitors ^38^.

***Regulation of hepatitis C virus replication by miR-122.*** One of the unique features of HCV replication is its requirement for the liver-specific microRNA miR-122, which enhances translation and replication through binding to the 5′ non-coding region (NCR) of HCV RNA ^39, 40^. Beyond contributing to the restricted tissue tropism of HCV for the liver, miR-122, in concert with Ago2, stabilizes the viral RNA ^41^ and prevents its decay by the exoribonuclease Xrn1 ^42^. Recent research suggests a role for another exoribonuclease, Xrn2, in miR-122-mediated prevention of viral RNA decay for certain HCV genotypes ^43^. However, Xrn2 restriction of HCV RNA replication is likely a marginal, indirect effect observed with only a few genotypes ^44^.

Recently, several reports brought novel insights to the roles and impacts of miR-122 sequestration by HCV on viral replication and liver homeostasis. By reducing the amount of viral genomes engaged in translation, miR-122 was recently shown to increase the fraction of viral RNAs available for replication, thus enhancing RNA replication and protein synthesis ^45^.

Another study recently reported that through the sequestration of miR-122, HCV RNA induces a global de-repression of miR-122 targets over the human transcriptome ^46^. The authors suggested that the miR-122 “sponge effect” by HCV RNA may contribute to unbalance liver homeostasis, hence favoring the development of liver cancers.

***Lipid peroxidation as repressor of hepatitis C virus replication.*** Unlike other viruses, HCV is sensitive to oxidative membrane damage, which usually occurs in stressed tissues. Lipid peroxidation affects the conformation of NS3-4A protease and NS5B, restricting HCV replication in cell culture and thus facilitating the long-term persistence of the virus within infected tissues ^47^.

The inability of non-adapted HCV strains or patient isolates to replicate in cell culture has strongly impacted HCV research over the last decades, thus limiting cell culture assays and interpretation to a single, non-adapted molecular clone, JFH-1. More importantly, the molecular mechanisms restricting replication of non-adapted strains in cell culture were unknown. Recently, a genome-wide gain-of-function screen found that the cytosolic lipid-binding protein SEC14L2 is an HCV host factor that allows detectable replication of diverse, non-cell culture-adapted HCV replicons and molecular clones in hepatoma cell lines which do not endogenously express SEC14L2 ^48^. Interestingly, the effect of SEC14L2 on viral replication was indirect, caused by an enhancement of vitamin E-mediated inhibition of lipid peroxidation. The discovery of SEC14L2 opens new avenues for the generation of non-adapted HCV cell culture systems, which could shed light on previously unknown aspects of HCV biology and genetic diversity.

***Novel insights into hepatitis C virus assembly.*** HCV particle assembly is a complex molecular process involving the recruitment of structural proteins and viral RNA at the assembly site, formation of the nucleocapsid, and the envelopment and maturation of the viral particle ^49^. As this process involves a considerable number of viral factors, host proteins, and lipid components, the molecular mechanisms and factors regulating this process are still not fully characterized, limiting the design of DAAs targeting the late steps of the HCV life cycle.

p7 is a small, hydrophobic viral protein that associates as multimeric complexes to form ion channels essential for viral assembly and release ^50^. Nevertheless, the precise molecular mechanism by which p7 regulates assembly still needs to be characterized. The recent structural resolution of the p7 ion channel has brought new insights into the potential mechanism of the action of p7 ^51^. p7 displays an unusual funnel-like architecture as well as a mechanism of cation selection mediated by two pairs of amino acids. Overall, this structure provides a clearer basis for p7-mediated cation conductance and insights on developing channel activity inhibition strategies.

Recently, another interesting report demonstrated that HCV’s hijacking of host innate immune signaling pathways enhances viral assembly. Indeed, indirect activation of the IκB kinase-α (IKK-α), a component of the NF-κB signaling cascade, by the 3′ NCR of HCV can activate a transcriptional program, leading to the induction of lipogenic genes and increased formation of core-associated lipid droplets ^52^. This provides a strong link between HCV-induced innate immune responses, lipid metabolism, and disturbance of liver lipid metabolism.

## B. Hepatitis C virus humanized mice models: moving forward

Owing to the narrow host tropism of HCV, restricted to humans and chimpanzees, HCV-host interactions *in vivo*, HCV-induced pathogenesis, and the development of anti-viral strategies have all been hindered by the lack of a tractable, cost-effective animal model for HCV infection ^53^. As murine hepatocytes do not support HCV entry and replication, human liver chimeric mice have been a prominent model to study HCV infection *in vivo* over the past few decades ^54^. However, this model is limited by donor-to-donor variability, high costs, and the immunodeficient background of the recipient mice. It was previously reported that CD81 and OCLN represent the minimal set of human factors required for HCV uptake into mouse cells *in vitro* ^17^. Recently, two successive studies reported the first genetically humanized mouse model through transient expression of human CD81 and OCLN or through stable expression of the four canonical HCV entry factors (i.e. CD81, SCARB1, CLDN1, and OCLN) ^55, 56^. Blunting of anti-viral signaling allowed low-level viral replication, *de novo* particle production, and completion of the viral life cycle *in vivo* ^55^. In the future, such a model, as well as its future refinements, will be of considerable use to further dissect HCV infection *in vivo* and evaluate vaccine strategies.

Furthermore, an immunodeficient mouse co-engrafted with human hepatocytes and human hematopoietic stem cells was found to support HCV infection and develop T-cell-specific responses ^57^. However, no viremia or B-cell responses could be observed, highlighting the improvements needed in this system. In the future, co-engrafted humanized mice able to mount improved immune responses will represent a unique platform for characterizing HCV infection *in vivo*, understanding critical immunological events regulating outcome of infection, and evaluating vaccine candidates.

## C. Immunity and pathogenesis

### 1. Recent insights into the front lines of defense against hepatitis C virus

Acute HCV infection is spontaneously cured in 20% to 30% of patients, whereas the great majority of them are unable to clear the virus and will develop a chronic infection in the face of an ongoing innate and adaptive immune response. However, the early immunological events regulating the outcome of infection are still poorly described.

***Innate immune responses to hepatitis C virus and viral countermeasures.*** The innate immune system represents the first line of defense of host cells against viral infections ^58^. The innate immune system detects viral infection largely through germline-encoded pattern recognition receptors (PRRs) such as Toll-like receptors (TLRs), the retinoic acid-inducible gene I-like receptors (RLRs), the nucleotide oligomerization domain-like receptors (NLRs), and cytosolic DNA sensors. Activation of these PRRs leads to the secretion of interferons (IFNs), key cytokines responsible for the establishment of an anti-viral state in cells ^59^.

During HCV infection, TLR3 recognizes HCV double-stranded RNA intermediates, thus inducing the production of inflammatory cytokines ^60^. In parallel, RIG-I recognizes the poly-U/UC tract of the HCV 3′ untranslated region (UTR), hence inducing the production of IFNs ^61^. However, recent evidence increasingly suggests that MDA-5 is also an important inducer of IFN production in HCV-infected cells ^62– 64^. Single-nucleotide polymorphisms of MDA-5 have been found to strongly correlate with the resolution of HCV infection, arguing a role for MDA-5 in the natural course of HCV infection ^65^.

It was conventionally thought that the plasmacytoid dendritic cells (pDCs), the main IFN producers of the immune system, were able to produce IFNs by recognizing infectious particles ^66^. However, a recent report showed that viral RNA-containing exosomes secreted by HCV-infected cells are the major immuno-stimulatory inducer of IFN secretion by pDCs via a TLR7-dependent mechanism ^67^. Exosomes also now appear to be an HCV propagation carrier, as it has been shown that exosome-associated viral RNA can induce a productive infectious cycle in non-infected hepatocytes ^68, 69^. However, in contrast to infection with viral particles, exosome-mediated infection is presumably less effective and thus raises the question of its biological significance *in vivo* ^70^.

To overcome the host innate immune system, the HCV NS3-4A protease can cleave the host adaptor proteins mitochondrial anti-viral-signaling protein (MAVS) and TIR-domain-containing adapter-inducing IFN-β (TRIF), thus inhibiting the RIG-I like receptors and TLRs-mediated type I and type III IFN signaling pathways within infected cells ^71– 73^. Recently, HCV NS4B was reported to block IFN production by disrupting STING interaction with MAVS and TBK1 ^74, 75^. These findings shed new light on the molecular mechanisms underlying the persistence of HCV infection.

***The control of hepatitis C virus infection by interferon λ.*** In HCV-infected patients, genetic variations in the IFNλ locus are associated with spontaneous viral clearance and type I IFN-based treatment success ^76– 79^. However, the molecular mechanisms underlying the close association between IFNλ polymorphisms and the clinical outcome of HCV infection remain poorly characterized.

A study recently highlighted an unsuspected molecular mechanism associating IFNλ3 polymorphism with HCV repression of the innate anti-viral response. An IFNλ3 mRNA carrying an unfavorable polymorphism was highly susceptible to AU-rich element-mediated decay and to binding of HCV-induced microRNAs, hence favoring repression of this IFNλ3 polymorphism ^80^. Overall, this study provides a potential explanation of why particular IFNλ3 alleles are better regulators of HCV infection.

In another study, laser capture microdissection was used to isolate HCV-infected primary human hepatocytes displaying different IFNλ genotypes. Interestingly, hepatocytes from donors with clinically less favorable IFNλ genotypes were more permissive to HCV infection and exhibited reduced anti-viral responses compared with cells from donors with favorable alleles ^81^. Hence, this represents additional, strong evidence that IFNλ alleles can predict the HCV permissiveness and innate immune responses of a particular host genetic background.

Overall, all these findings highlight the importance of host genetic factors and of inducers of the innate immune response in determining the early events of infection. In the future, the combination of high-throughput transcriptomic and single-cell technologies with relevant *in vivo* experimental models could help to better characterize the molecular and immunological factors regulating the early events of infection and, ultimately, outcome of HCV infection.

### 2. Adaptive immunity: impact of T-cell dysfunctions in chronic hepatitis C virus infection

Failure of the innate immune system to control early events of infection induces the development of an adaptive immune response against HCV, highlighted by the generation of an HCV-specific T-cell response and the production of HCV neutralizing antibodies ^82^. However, the immune mechanisms underlying the failure of the cytotoxic and humoral responses in resorbing viral infection and leading to a state of chronic infection are not well understood.

Recently, several reports shed light on the impacts of T-cell dysfunction during HCV infection as well as on the molecular mechanisms contributing to such dysfunctions, which can favor the inhibition of long-term adaptive immune responses and thus the maintenance of chronic infection.

During chronic infection, continuous antigenic stimulation can enhance the expression of inhibitory receptors on cytotoxic T-cells (CTLs), leading to impaired CTL functions. A recent report supported this argument in identifying a novel inhibitory receptor, prostaglandin E2, overexpressed on the CTL surface during lymphocytic choriomeningitis virus infection ^83^. Blocking of prostaglandin E2 and programmed cell death 1 signaling improved CTL responses and favored better immune control of chronic viral infection. This evidence strongly suggests that similar mechanisms are at play during HCV infection and likely contribute to sustain chronic infection. Another study also recently highlighted how T-cell function may be impaired during HCV infection. HCV E2 protein and a short E2-coding RNA fragment were found to inhibit distal and proximal T-cell receptor-mediated signaling, respectively ^84^. By affecting T-cell activation, HCV E2 protein and RNA may contribute to a global state of T-cell dysfunction and impaired adaptive immune responses favoring chronic infection. Consistently, CD8 ^+^ T-cell responses have been shown to be restored in patients following DAA-based, IFN-free therapy. The suppression of viral replication could disrupt the global state of T-cell dysfunction and reinstate T-cell function, which might be critical for the success of the therapy ^85^.

Interesting findings have also been reported regarding the impact of maternal immune tolerance during pregnancy on HCV-specific T-cell functions. Impaired T-cell responses induced by pregnancy have been shown to limit T-cell-mediated selective pressure on HLA-I epitope, hence stimulating the loss of escape mutations and the emergence of fitter virus ^86^. Indeed, T-cell selective pressure was shown to be restored after childbirth along with the predominance of escape mutations. This suggests that maternal immune tolerance allows viruses with enhanced fitness to be vertically transmitted into a new host.

T-cell function can also be seriously impaired by extensive regulatory T (Treg) cell expansion during HCV infection. Indeed, a recent report analyzed the HCV-specific T-cell response following HCV challenge of non-human primates previously infected with a subinfectious dose of HCV. Although subinfection induced the development of an HCV-specific T-cell response, subsequent challenge led to expansion of Treg cells that suppressed an effective T-cell and recall response ^87^. Hence, individuals who were repeatedly exposed to HCV may be more prone to develop chronicity through exposure-induced immune suppression and strong Treg cell expansion.

## D. Curing and preventing hepatitis C: where are we going?

### 1. Direct-acting anti-viral agents: promises and challenges

Over the last 3 years, a combination of DAAs involving NS3-4A inhibitors, NS5A inhibitors, and NS5B nucleos(t)ide or non-nucleos(t)ide inhibitors has demonstrated their strong potency to induce a sustainable virologic response close to 90% to 100% against the most prevalent HCV genotypes ^2, 88^, thus allowing HCV patients to be cured. However, several challenges will likely appear in the future, as most of these drugs have a low barrier to resistance, with the exception of NS5B nucleos(t)ide inhibitors. Indeed, resistance-associated mutations to several DAAs have already been characterized in NS3-4A, NS5A, and NS5B as well as natural polymorphisms observed in certain genotypes and subtypes ^3^.

However, strategies are currently being developed to increase the resistance barrier of DAAs. Daclatasvir (DCV) interferes with NS5A functions and is a very potent HCV inhibitor, but mutations within NS5A can arise fairly easily, rendering HCV resistant to DCV’s anti-viral activity ^89^. However, recent findings demonstrated that this resistance is overcome when DCV is used in combination with an NS5A inhibitor analogue, commonly inactive against both wild-type and resistance NS5A variants. This synergistic effect underscores the importance of inter-protein communication between NS5A molecules in the mechanisms of action of DCV. Moreover, this finding emphasizes that progress in understanding viral protein functions is critical to enhance resistance barriers of DAAs and to develop rational DAA combination therapy for effective clinical treatment ^90^.

Moreover, another critical challenge lies in the fact that curing HCV patients does not mean they are cured of liver disease or protected against the development of potential HCC. However, our understanding of the HCV-induced mechanisms leading to liver disease and cancer remains somewhat limited. Hence, a strong emphasis on these mechanisms will be required in the upcoming years to develop original therapies preventing the development of such diseases and face the need of protecting HCV-cured patients against them.

The appearance of HCC in chronically infected patients is likely to be stimulated by the immune tolerance induced by continuous antigen stimulation and T-cell dysfunction. Hence, the prevention of HCV-induced HCC requires an improved understanding of the immunological events favoring both the maintenance of HCV chronic infection and the appearance of HCV-induced liver cancers. A better profiling of the dysfunctions of the adaptive immune responses during HCV infection could provide innovative immunotherapy strategies that could reduce the risk of HCC in chronically infected patients or HCV-cured patients. Moreover, the recent success of immunotherapies against lung and melanoma cancers ^91^ also highlights the need for improved knowledge about liver cancer immune evasion mechanisms, which could open avenues for treating HCV-free patients who develop HCV-induced HCC.

### 2. The quest for an effective hepatitis C virus vaccine

Despite the clinical efficacy of the DAAs, the great majority of HCV-infected patients worldwide do not have access to these treatments because of their high cost. One solution lies in the development and production of novel, cost-effective DAAs or antibodies that could cure HCV patients. This possibility is supported by data from recent studies that demonstrated that passively administered anti-HCV envelope ^92^ and anti-CLDN1 antibodies can cure human liver chimeric mice of chronic HCV infection ^93^.

In parallel, a cost-effective, prophylactic, and therapeutic HCV vaccine is still urgently needed to significantly impact the number of HCV cases worldwide. However, such research has been extremely challenging over the past two decades. The incomplete knowledge of the adaptive immune response to HCV and exposure-induced immune suppression limits the design of T-cell-based vaccines. Additionally, the high E1E2 genetic variability has hampered the generation of broadly potent immunogens. Several past and recent reports have found that the generation and use of broadly neutralizing antibodies (bNAbs) targeting E1E2 can lead to efficient, pan-genotypic neutralization of HCV *in vitro* ^94, 95^ and even HCV clearance *in vivo* ^92^. However, such bNAbs are usually poorly induced in chronically infected patients. Hence, the generation of immunogens able to trigger an effective bNAb response in patients has emerged as a considerable barrier to the generation of a potent HCV vaccine. An important obstacle in the development of such immunogens likely lies in our poor understanding of the structural and functional basis of HCV neutralization, E1E2 conformational plasticity, and epitope accessibility. The recent structural resolution of an E2core structure ^25, 26^ represents a considerable advancement for elucidating the mechanisms of HCV neutralization. However, on the surface of viral particles, it is likely that E2 takes on other conformational states, as E2core is only a partial structure and E2 conformation is dependent on its association with E1. In the future, novel insights into E1E2 structures and conformational changes will provide important avenues for the generation of an effective vaccine.
